# Level and Determinants of Knowledge of Symptomatic Knee Osteoarthritis among Railway Workers in Malaysia

**DOI:** 10.1155/2014/370273

**Published:** 2014-02-19

**Authors:** Kurubaran Ganasegeran, J. Michael Menke, Vasudeva Murthy Challakere Ramaswamy, Rizal Abdul Manaf, Aied M. Alabsi, Sami Abdo Radman Al-Dubai

**Affiliations:** ^1^International Medical School, Management and Science University (MSU), Shah Alam 40100 Selangor, Malaysia; ^2^International Medical University (IMU), 57000 Bukit Jalil, Kuala Lumpur, Malaysia; ^3^Department of Pathology, International Medical University (IMU), 57000 Bukit Jalil, Kuala Lumpur, Malaysia; ^4^Community Health Department, Faculty of Medicine, Universiti Kebangsaan Malaysia (UKM), Cheras, 56000 Kuala Lumpur, Malaysia; ^5^Department Oral Biology and Biomedical Science, Faculty of Dentistry, University of Malaya (UM), 50603 Petaling Jaya, Kuala Lumpur, Malaysia; ^6^Department of Community Medicine, International Medical University (IMU), 57000 Bukit Jalil, Kuala Lumpur, Malaysia

## Abstract

*Background*. Symptomatic knee osteoarthritis, an ancient malady greatly impairing modern population quality of life, has stimulated global attention to find effective modes of prevention and intervention. *Purpose*. This study aimed to assess factors affecting knowledge of symptomatic knee osteoarthritis (knee OA) among Malaysian railway workers. *Methods*. A cross-sectional study was conducted among 513 railway workers involving eight major states within Peninsular Malaysia using population-based sampling. The assessment instrument was a face-validated, prepiloted, self-administered instrument with sociodemographics and knowledge items on knee OA. *Results*. Mean (±SD) age of the respondents was 41.4 (±10.7), with the majority aged 50 years or older (34.9%). Of the total respondents, 53.6% had low levels of knowledge of knee OA disease. Multivariate analysis found that four demographic predictors, age ≥50 years, family history of knee OA, self-awareness, and clinical diagnosis of the disease entity, were significantly associated with knowledge scores. *Conclusion*. The finding of a low level knee OA knowledge among Malaysian railway workers points to an urgent need for massive information to be disseminated among the workers at risk to foster primary prevention and self-care.

## 1. Introduction

Ancient descriptions often referred to musculoskeletal pain and dysfunction as “rheumatism” [1], but paleopathological evidence of excavated skeletons that illustrated degenerative and focal lesions within narrowed joint spaces prompted a more specific and apt term of “osteoarthritis,” coined by John Spender in 1886 [2, 3]. Later, OA and associated clinical manifestations were shown to be distinct from rheumatoid arthritis (RA) in pathogenesis and epidemiology [2].

Symptomatic knee OA is currently the fourth leading cause of disability worldwide, [4] with an estimated prevalence of 70–80% in the population aged 55 years and older [5]. An ageing Asia with longer lifespan suggests ever-greater numbers of knee OA, among other chronic conditions [6]. The point prevalence of knee OA in Malaysia today is estimated to be 10–20% of the total adult population [7].

The diagnosis of knee OA lies in both clinical and radiological terms. It is a clinical disease consisting of subjective symptoms of joint pain on loading and bony swelling, objective physical examination of knee stiffness, and deformity or crepitations, along with supplementary radiographic findings [8, 9]. The Kellgren-Lawrence (K-L) grading scale based on radiographic X-ray has been the standard and acceptable quantification tool to assess knee OA severity [9]. The K-L scoring ranges from 0 to 4 where 0 is normal radiograph; 1 is doubtful pathology; 2 is minimal osteophytes with possible narrowing, cysts, and sclerosis; 3 is moderate with definite osteophytes and moderate joint space narrowing; and 4 is severe with large osteophytes and definite joint space narrowing [10]. The grade of ≥2 is classified as osteoarthritis [11].

The Framingham Knee Osteoarthritis Study called knee OA a “disease of the older age” [12]. Disease etiology is multifactorial that includes generalized constitutional (female gender, obesity) [13, 14] and hormonal (postmenopausal state) factors [15, 16] and local adverse mechanical insults to the knee joint (joint trauma, occupational mechanical loading of the knee joints, and sports and recreational abuse) [13, 14, 17, 18]. Geographical factors like climate change had no predisposition to knee OA [19].

Pathognomonic of knee OA includes significant pain over knee joints associated with varying degrees of functional limitation and reduced quality of life [20]. Other clinical manifestations of knee OA include joint stiffness or numbness with inflammatory signs like swelling, warmth, redness, and crepitus of the knee due to effusions which further exacerbates instability symptoms like “clicking” or “locking” of knee joints causing restricted movement and quadriceps muscle weakness [13, 14, 18].

A major threat to full employment at older ages is the incapacity arising from symptomatic knee OA [21]. An ageing workforce will contain ever-greater proportion of workers with knee joint pathology [21]. Occupational related activities like walking, sitting, standing, kneeling, squatting, climbing, and heavy lifting and much of self-care will undoubtedly be difficult and uncomfortable with knee OA [21]. Modern research has prompted global economic evaluation as a result of “employment retrenchment,” “job absenteeism,” “job quitting,” “sick leave,” “work participation,” “work changes,” “work adaptations,” and “work transitions” due to knee OA functional limitations among workers [22].

A sample of 254 patients with knee OA from Italy reported as 2.4% for discontinuing work, 2% changed the type of work, and 22% with regular knee OA-related sick leave [23]. Similarly, Sayre et al. (2010) [24] investigated “employment reduction” due to OA among subjects from British Columbia and found that 36% of the subjects with knee OA had quit work entirely and 13% had reduced working hours.

The OA Research Society International (OARSI) reported that treatments like acetaminophen, nonselective and selective oral nonsteroidal anti-inflammatory drugs (NSAIDs), topical NSAIDs and capsaicin, intra-articular injections of corticosteroids or hyaluronans, glucosamine, and/or chondroitin sulphate may be used for symptom relief, while glucosamine sulphate, chondroitin sulphate, and/or diacerein are administered for structure-modifying effects, and weak opioids or narcotic analgesics are used for the relief of refractory pain [25]. Nonpharmacological management like applying hot or cold compression to the knee joint has been shown to improve knee pain [26–31]. Supportive footwear has been suggested as a preventive measure for knee OA [30, 31] and recently was postulated to improve knee loading among knee OA patients [32]. Surgical options like total knee arthroplasty are made available for severe knee OA [17].

Rising prevalence rates and longer lifespan indicate a pending global public health crisis, yet only sparse information for identifying and preparing for it still exists in most developed and developing nations. Despite detailed published accounts of self-management techniques and knee OA prevention, for example, the NICE Guidelines and the Arthritis Foundations' National Public Health Agenda, misconceptions, lack of preventive knowledge, and maladaptive sociocultural beliefs among caregivers and general population in different regions pose substantial barriers towards preventive efforts [33].

To the best of our knowledge, this study was the first population-based study that assessed knowledge of symptomatic knee OA among railway workers. Data on prevalence rates of knee OA among railway workers was rather scarce from both developed and developing countries. Railway workers with diverse occupational traits from both manual and administrative sectors involving locomotive engine pilots or drivers, railroad yardworkers, trackmen, maintenance workers, signalers, station masters, shunters, and office workers were predominantly classified as high strained workers because of their prolonged exposure to rigid protocols and limited rests [34]. Crushed rock ballast covered on railroad yards and tracks creates irregular stance and motions [35]. Railroad yardworkers, shunters, signalers, and trackmen as well as strenuous jobs that demand prolonged improper standing of abnormal stance [18, 35–38], irregular walking postures, kneeling, or squatting postures for railroad track alignments [39, 40] were at higher risk of knee OA due increased stresses at the knee joint as a consequence of greater torsional loading on the menisci [35, 41]. Locomotive engine pilots, train drivers, shunters, and office administrative workers sitting with frequent short length seats in prolonged flexion of the knees suffer increased stress load on the knee joint, prompting an increased risk of knee OA [30].

Occupational health in Malaysia is regulated mainly by the Occupational Safety and Health Act 1994. The responsibilities of safety and health at the workplace lie with those who create the risk and with those who work with the risk. It stresses on self-regulation and participation and cooperation of workers. The Department of Occupational Safety and Health (DOSH) is a department under the Ministry of Human Resources which is responsible for standard setting of relevant legislation and code of practice and guidelines as well as enforcement through the regional branch office in each state in the country [42]. Despite these legislations and guidelines, workplace injuries and occupational health disease that demands increased workers compensation remained high in Malaysia [43]. The “Osteoarthritis Public Health Agenda Policy and Communications White Paper” drafted nine priority policies of urgent tasks [44]. The third priority policy, “increase early access to evidence-based interventions,” highlighted the need to promote awareness and knowledge of knee OA among general population and caregivers. As a part of global initiative to foster primary prevention and promote understanding, this study assessed levels of knee OA knowledge in a population of Malaysian railway workers—a population of workers at higher risk of developing knee OA.

## 2. Materials and Methods

### 2.1. Ethics Statement

This study complied with the guidelines in the Declaration of Helsinki. Study protocol was approved by the affiliated institutional ethical committee. Following institutional review board, the Cooperative Society *Keretapi Tanah Melayu Berhad* (KTMB), being representative of all railway workers in Malaysia, granted permission and final approval. Objectives and benefits of the study were explained in written form and attached to the questionnaires for respondent approval. Respondents were assured that information would be confidential and participation would not affect their working status. A written consent was obtained from those who agreed to participate.

### 2.2. Study Design and Population

This descriptive cross-sectional study was conducted in the month of April 2012 among all workers from “*Keretapi Tanah Melayu Berhad*” (KTMB), the country's largest railway network provider in Peninsular Malaysia. The organization encompassed different job traits (e.g., mechanical, electrical, carpentry, administration, engineering, etc.) comprising of both blue-collar and white-collar workers, therefore making the population being studied representative of the country's main human work force with resources from both skilled and semiskilled sectors. The study recruited all 729 workers registered to the Cooperative Workers Society (KTMB) using population, based sampling involving eight states within Peninsular Malaysia: 294 workers from Kuala Lumpur, 108 from Selangor, 104 from Perak, 91 from Johor, 64 from Kelantan, 30 from Negeri Sembilan, 20 from Kedah, and 18 from Penang. After receiving relevant permissions from the office, we obtained the respondents' postal address. Respondents were approached through a postal survey and were requested to return the completed questionnaires to the Cooperative Workers Society's main office in Kuala Lumpur within a month, after which we recollected all data from the office. Respondents less than 20 years of age were excluded along with those who refused participation.

### 2.3. Study Instruments

We designed a 36-item self-administered questionnaire regarding knowledge of knee OA from literatures and proven hypotheses. Validated items on risk factors [14, 16, 19, 21, 30, 36, 37, 39–41, 45–47], signs and symptoms [8, 13, 14, 18, 48], disabilities [8, 14, 21], and prevention and management [17, 22, 26–32] extracted from previous studies were conjugated as knowledge scales in the present study. The questionnaire was first piloted among 50 railway workers and slightly modified according to their comments. An expert orthopedic surgeon conducted face validation to ensure the knowledge scale conformed to established knee OA disease criteria. The final questionnaire was comprised of two domains: sociodemographic data and knee OA knowledge. Sociodemographic questions included gender, age, education level, type of occupation, awareness of knee OA as a disease and signs and symptoms, awareness of any immediate family members with knee OA, and whether the respondent has ever been diagnosed with knee OA. Knowledge was assessed in five subsections: (1) risk factors (11 items), (2) signs and symptoms (10 items), (3) disabilities (7 items), (4) prevention (4 items), and (5) management (4 items). The questions for each subscale were structured as follows. Regarding knowledge of knee OA, consider the following. (1) Do you think that the following are risk factors of knee OA? (2) Do you know if the following are signs and symptoms of knee OA? (3) Do you think that knee OA causes the following disabilities? (4) Do you think that the following measures help to prevent knee OA? (5) Do you think that the following measures help to relieve knee OA symptoms? Response options were “Yes,” “No,” and “I do not know.” The questionnaire was administered in the local language, Malay.

### 2.4. Statistical Analysis

Analysis was performed using Statistical Package of Social Sciences (SPSS) (version 16.0, IBM, Armonk, NY). For each item in the knowledge part, the correct answer was coded (1), while the incorrect answer or “I do not know” was coded (0). Scores of knowledge items were summed to obtain the total knowledge score on knee OA (minimum = 0 and maximum = 36). A higher score represents better knowledge. A high knowledge level was classified based on median cut-off point of the total score (≥21). Descriptive analysis was performed for all variables in this study. Knowledge scores were expressed as mean and standard deviations. Test of normality was performed for the total knowledge score. *t*-test and ANOVA test were applied to compare knowledge across sociodemographic variables. In case of ANOVA, post hoc test was used to determine where the significant difference was. Multiple linear regression analysis using “backward elimination” was employed to obtain significant factors associated with knee OA knowledge. The accepted level of significance was set below 0.05 (*P* < 0.05). Multicollinearity was checked between independent variables.

## 3. Result

### 3.1. Sociodemographic Characteristics of Respondents

A total of 513 railway workers with a response rate of (70.3%) consented to participate in the survey. The mean (±SD) age of respondents was 41.4 (±10.7) years and the majority aged 50 years or older (34.9%). The majority of respondents were males (74.9%), white-collar workers (52.0%), and secondary educated (69.1%). Less than half (40.2%) of the entire sample had an immediate family member with the condition. One hundred five (20.5%) of total respondents reported having knee OA diagnosed clinically by a doctor (Table 1).

### 3.2. Awareness and Knowledge of Knee Osteoarthritis among Respondents

Most of the respondents expressed awareness of knee OA as a disease entity (60.2%). Based on median cut-off points of knowledge score, 53.6% of the respondents had low levels of knowledge.  Table 2 exhibits individual item scores of knee OA.

### 3.3. Association between Knowledge of Knee Osteoarthritis and Sociodemographic Variables

Table 3 shows the associations between sociodemographic factors and knowledge of knee OA among respondents. Male respondents displayed higher knowledge score (18.8 ± 10.5) compared to females (15.0 ± 11.4, *P* < 0.001). There was a significant association between age and knowledge (*P* < 0.001); a post hoc test revealed that respondents aged 50 years or more had higher knowledge score (21.7 ± 9.1) compared to respondents aged 35–49 years old (18.2 ± 10.9) and compared to 20–34 years (13.3 ± 10.9; *P* = 0.003, *P* < 0.001, resp.). There was a significant association between education level and knee OA knowledge (*P* < 0.001) and post hoc tests showed that respondents with tertiary education (20.8 ± 8.8) showed higher knowledge score than those with secondary education (16.8 ± 11.5, *P* < 0.001). Blue-collar workers exhibited higher knowledge score (19.8 ± 10.3) than white-collar workers (16.2 ± 11.1; *P* < 0.001). Those respondents aware of knee OA had better overall knowledge score of knee OA (24.5 ± 6.2) when compared to those generally unaware of the condition (7.9 ± 8.5; *P* < 0.001). Respondents having immediate family members with knee OA had higher knowledge score (25.5 ± 5.7) compared to respondents without a family history of knee OA (12.8 ± 10.5; *P* < 0.001). Respondents who were clinically diagnosed for knee OA showed higher knowledge score (25.7 ± 5.6) compared to those without such medical condition (15.9 ± 11.0; *P* < 0.001).

### 3.4. Factors Associated with Knowledge of Symptomatic Knee Osteoarthritis among Respondents by Multiple Linear Regression

Table 4 exhibits factors associated with knowledge of knee OA among Malaysian railway workers. Respondents aged 50 years or older had on the average 1.7 (95% CI 0.1–3.2) higher score in OA knowledge compared to respondents in the 20–34 age group (*P* = 0.035). Respondents being aware of knee OA had on the average 12.5 (95% CI 11.0–13.9) higher knowledge score compared to respondents being unaware of the condition (*P* < 0.001). Similarly, respondents with an immediate family member with knee OA averaged a 5.5 (95% CI 4.1–6.9) higher knowledge score than respondents whose family members did not have the condition (*P* < 0.001). Respondents diagnosed clinically of knee OA averaged 2.06 (95% CI 0.5–3.7) higher knowledge score in comparison to respondents without knee OA (*P* = 0.011).

## 4. Discussion

### 4.1. Core Summary Findings

This cross-sectional study was aimed at determining factors affecting knowledge of symptomatic knee OA among Malaysian railway workers. Of the 513 railway men surveyed, 53.6% reported low levels of knowledge. Our final regression model yielded four variables significantly influencing knee OA knowledge in this group: respondents over 50 years old, respondents previously aware of knee OA, immediate family members with knee OA, and respondents with knee OA diagnosed clinically by a doctor.

### 4.2. Comparison with Existing Literature

Data on risk factors, clinical manifestations, and available treatment options was well documented in numerous epidemiological studies and clinical trials [6, 49–51]. Our study challenged the current tenets and dogmas that only pathological, radiological, and clinical investigations may elicit awareness and knowledge of symptomatic knee OA. Hypotheses tested in previous studies were conjugated as knowledge scales in the present study to understand the lay population's perception of this disease entity to promote primary prevention. This study was the first to explore knowledge of symptomatic knee OA in the general population, with particular focus among a specific occupational group.

Our sample demonstrated a broad spectrum of preventive knowledge, including self-management techniques for symptomatic relief. A majority reported that avoidance of excessive and prolonged knee weight bearing activities and optimal body weight are the gold standards for prevention. However, the notion that exercise accelerates quadriceps muscle strength and helps to prevent knee OA [52] was less understood in our sample. Respondents in our study agreed that pain killers and anti-inflammatory drugs would prompt symptomatic relief, inconsistent with a recent report from the UK [33].

Female gender and ages greater than 50 years old were at higher risk for developing knee OA [53]. Prevalence of knee OA was higher among Asian women as compared to Asian men [6]. The Beijing OA study concluded that symptomatic knee OA among Chinese women aged 50 years or older was 2.9 times higher than men [54], suggesting hormonal factors affecting women during menopausal phase accelerates the development of knee OA [55]. Despite greater susceptibility in women, female respondents in this study exhibited significantly lower knowledge of knee OA than their male counterparts. Similarly, this study showed significant differences between age groups with respect to knowledge, with respondents aged over 50 years exhibiting greater understanding of knee OA.

Educational attainment was linked to pain and disability in osteoarthritis [56]; indeed a higher educational level was more predictive for disease progression [57]. Behavioral risks that influenced health-seeking behavior, access, and utilization of health services for early interventions were associated with higher educational level [58]. A higher knowledge of knee OA was found for respondents with tertiary education over those with a primary or secondary education. Andrianakos et al. (2006) [53] found similar findings in the Greek population.

Repetitive strain at tibiofemoral or patellofemoral joints during work is associated with an increased risk of knee OA [59, 60]. Knee flexion for squatting and kneeling among manual workers are routines, thus predisposing greater risk for development of symptomatic knee OA [61, 62]. Prevalence rates corresponded with knee OA knowledge. This study found significant association between knowledge of knee OA and occupational level, albeit blue-collar workers portrayed higher knowledge than white-collar workers.

Family history reflects a higher genetic susceptibility and shares environmental or behavioral factors of disease [63]. Knee OA was less likely to be genetically inherited, but more likely to be caused by shared environmental factors [64]. Not surprisingly, the respondents who had immediate family members with symptomatic knee OA exhibited higher knowledge than those without family history. In addition, respondents being aware of knee OA or have been clinically diagnosed for the disease exhibited a significantly higher knowledge level of the disease. Higher knowledge in families that has been explained [65] by family member experiences of a disease induces feelings of vulnerability through vicarious learning. From the results above, it may be inferred that inculcating awareness through public health education is an important primary preventive tool to increase knowledge of knee OA disease. Higher knowledge level among workers with knee OA or those with a family history of knee OA in comparison to workers without disease exposure indicates that education is a typical way to increase the awareness of a particular disease.

### 4.3. Study Limitations

The following limitations should be acknowledged in this study. This is the first population-based study to measure and self-report knowledge of symptomatic knee OA among workers. Exclusive reliance upon knowledge scales used here may pose correlation inflations. The cross-sectional nature of this study could not establish the causal relationships. The issue of measurement variation could be another limitation in this study.

### 4.4. Implications and Future Direction

This study initiated in-depth insights of symptomatic knee OA knowledge among the general population, stimulating a national public health agenda for prevention of knee OA and related problems. Access to information, education, and social support is vital [66]. Behavioral modification techniques combined with education facilitate self-efficacy of knee OA [67]. Policies for preventive efforts might be implemented in community health settings and major health care facilities around the country.

## 5. Conclusion

The results from this cross-sectional study impact important public health decisions, given that respondents over 50 years, with a family history, self-awareness, and a knee OA diagnosis, showed better knowledge of symptomatic knee OA as shown in the multivariate analysis. In general, Malaysian railway workers projected low levels of knowledge regarding symptomatic knee OA, demonstrating an alarming ignorance on the subject, suggesting the need for more and better information to be disseminated among the railway workers.

## Figures and Tables

**Table 1:** Sociodemographic characteristics of the respondents (*n* = 513).

Characteristic	*N*	%
Gender		
Male	384	74.9
Female	129	25.1
Age group (years)		
20–34	157	30.6
35–49	177	34.5
≥50	179	34.9
Education level		
Primary	11	2.1
Secondary	354	69.1
Tertiary	148	28.8
Occupation		
Blue-collar	246	48.0
White-collar	267	52.0
Awareness of knee osteoarthritis as a disease entity		
Yes	309	60.2
No	204	39.8
Immediate family members with knee osteoarthritis		
Yes	207	40.4
No	306	59.6
Diagnosed clinically to have knee osteoarthritis		
Yes	105	20.5
No	408	79.5

**Table 2:** Knowledge of knee osteoarthritis among respondents (*n* = 513).

Statement	Correct answer	*n* (%)
Risk factors		
Female gender	Yes	164 (32.0)
Postmenopausal women are more likely to have osteoporosis, not osteoarthritis	No	346 (67.4)
Age 50 years or older	Yes	236 (46.0)
Obesity is not a risk factor	No	303 (59.1)
Previous knee injury	Yes	325 (63.4)
Sports and leisure time physical activities	Yes	262 (51.1)
Repetitive strain injury	Yes	214 (41.7)
Working in a kneeling or squatting position	Yes	267 (52.0)
Climate change has no risks	Yes	162 (31.6)
Sitting improperly for long time	Yes	196 (38.2)
Standing improperly for long time	Yes	217 (42.3)
Signs and symptoms		
Knee pain	Yes	370 (72.1)
Stiffness of the knee during the night	No	265 (51.7)
Feeling of increased warmth in the knee	Yes	208 (40.5)
Redness of the knee	Yes	190 (37.0)
“Clicking” or “catching” of the knee	Yes	256 (49.9)
“Locking” of the knee or unable to fully straighten	Yes	271 (52.8)
Weakness of muscles of the thigh	Yes	249 (48.5)
No swelling of the knee	No	281 (54.8)
Numbness or tingling sensation of the knee	Yes	232 (45.2)
Pain when touched or pressed the knee	Yes	272 (53.0)
Disabilities		
Bed ridden rather than difficulty rising from bed	No	303 (59.1)
Difficulty putting on stockings	Yes	255 (49.7)
Difficulty rising from sitting	Yes	328 (63.9)
Difficulty bending to the floor involves osteoarthritis of the hip, not knee	No	326 (63.5)
Difficulty kneeling	Yes	334 (65.1)
Difficulty squatting	Yes	332 (64.7)
Movements are totally impaired	No	186 (36.3)
Prevention		
Regular exercises	Yes	255 (49.7)
Optimal body weight is not necessary for prevention	No	343 (66.9)
Avoiding excessive and prolonged weight bearing to the knees during work	Yes	347 (67.6)
Supportive footwear	Yes	222 (43.3)
Management		
Pain killers and anti-inflammatory drugs help to relieve osteoarthritis pain temporarily	Yes	269 (52.4)
Intra-articular steroid injections help to relieve severe symptoms temporarily	Yes	212 (41.3)
Knee replacement surgery is indicated for severe osteoarthritis	Yes	97 (18.9)
Hot or cold packs to the knees help to resolve osteoarthritis symptoms temporarily	Yes	95 (18.5)

**Table 3:** Association between knowledge of knee osteoarthritis and socio-demographic variables among respondents (*n* = 513).

Characteristic	Mean (SD)	*P* value
Gender		
Male	18.8 (10.5)	
Female	15.0 (11.4)	<0.001
Age group (years)		
20–34	13.3 (10.9)	
35–49	18.2 (10.9)	
≥50	21.7 (9.1)	<0.001
Education level		
Primary	13.4 (8.3)	
Secondary	16.8 (11.5)	
Tertiary	20.8 (8.8)	<0.001
Occupation		
Blue-collar	19.8 (10.3)	
White-collar	16.2 (11.1)	<0.001
Awareness of knee osteoarthritis as a disease entity		
Yes	24.5 (6.2)	
No	7.9 (8.5)	<0.001
Immediate family members with knee osteoarthritis		
Yes	25.5 (5.7)	
No	12.8 (10.5)	<0.001
Diagnosed clinically to have knee osteoarthritis		
Yes	25.7 (5.6)	
No	15.9 (11.0)	<0.001

**Table 4:** Factors associated with knowledge of symptomatic knee osteoarthritis among respondents by multiple linear regression (*n* = 513).

	B	SE	Beta	*P* value	95% CI	
					Lower	Upper
Age (35–49 years)	1.3	0.7	0.1	0.078	−0.1	2.8
Age (≥50 years)	1.7	0.8	0.1	0.035	0.1	3.2
Education (tertiary)	1.3	0.7	0.1	0.056	0.0	2.6
Type of work (blue-collar)	1.0	0.6	0.1	0.083	−0.1	2.2
Awareness of knee osteoarthritis as a disease entity	12.5	0.7	0.6	<0.001	11.0	13.9
Having family members with knee osteoarthritis	5.5	0.7	0.2	<0.001	4.1	6.9
Diagnosed clinically to have knee osteoarthritis	2.1	0.8	0.1	0.011	0.5	3.7

*The reference group for gender is “female”; for age is “20–34 years”; for education level is “primary”; for occupation is “white-collar”; for all other variables is “no.”

**B: unstandardized coefficients, SE: standard error, Beta: standardized coefficients, CI: confidence intervals.

## Abbreviations

OA: Osteoarthritis
RA: Rheumatoid arthritis
SD: Standard deviation.
